# A Qualitative and Quantitative Analysis of Protein Substitution in Human Burn Wounds

**Published:** 2009-09-29

**Authors:** Hamid Joneidi-Jafari, Adrien Daigeler, Joerg Hauser, Hans-Ulrich Steinau, Walter Klatte, Ulrich Fischer, Marcus Lehnhardt

**Affiliations:** ^a^ Department of Surgery, BG-University Hospital Bergmannsheil, Ruhr University Bochum, Germany; ^b^ Department of Plastic Surgery, Burn Center, Handsurgery, Sarcoma Reference Center, BG-University Hospital Bergmannsheil, Ruhr University Bochum, Germany; ^c^ Biotest Pharma GmbH, Dreieich, Germany; ^4^ Department of Hand, Plastic and Reconstructive Surgery, Burn Centre, BG Trauma Centre, Ludwigshafen, Germany.

## Abstract

**Objective:** In major burn wounds of more than 15% total burn surface area mediator-associated reactions lead to capillary leak resulting in critical condition. Little is known about the efficiency of protein substitution. We quantified and qualified the systemic and local protein loss in burn patients during protein substitution, comparing fresh frozen plasma and the human serum protein solution Biseko. **Methods:** In 40 patients suffering from second-degree burn wounds with the total burn surface area between 20% and 60%, immediately after admission a defined wound surface area was enclosed with in a wound chamber. Wound fluid and serum samples were collected in 8 hour intervals for 2 days. Samples were analyzed for total protein, albumin, immunoglobulins -A, -G, -M, clotting parameters, c-reactive protein, and white blood cells. Protein substitution started 24 hour posttrauma. In a randomized pattern, patients received equal volumes of fresh frozen plasma or Biseko. **Results:** Total protein and albumin accumulated in high concentrations in wound fluid. With beginning of fresh frozen plasma substitution on day 2 posttrauma, serum total protein (1.7 g–3.9 g) and albumin (1.3 g–3.4 g) concentrations increased. Substitution of Biseko resulted in a stronger increase (serum total protein 1.8 g to 4.5 g, albumin 0.9 g to 3.4 g). Wound fluid concentrations revealed similar change patterns. Immunoglobulins showed higher serum levels in the Biseko group. C-reactive protein and white blood cell values indicated a lower immunological reaction in the Biseko group. **Conclusions:** Substitution of human protein solutions such as Biseko can result in significantly higher serum protein and albumin concentrations as well as lower infection parameters. Higher serum immunoglobulins could help to decrease potential immunodeficiency.

Severe burn injuries of more than 15% total body surface area (TBSA) result in burn disease that is characterized by electrolyte imbalance, loss of proteins followed by fluid dysregulations, circulatory insufficiency, and immunodeficiencies.^1^,^2^ These dysregulations arising from capillary leakage (CL) potentially lead to systemic inflammatory response syndrome, disseminated intravascular coagulation, hypermetabolism, hypoxia, and catabolism, and potentially result in multiorgan distress syndrome.^3^,^4^

Capillary leakage is known to appear approximately 24 hours posttrauma and is not restricted to the burned area. The intravascular proteins extravasate mainly within the first 24 hours and include the important albumin (AL) fraction. The protein shift leads to an increased extravascular oncontic pressure followed by intravascular hypovolemia, blood thickening, and edema.^1^,^5^,^6^ The enormous loss of intravascular proteins can be considered as a main reason for volume shifting. Both volume shifting and the resulting hypovolemia are known to be the primary cause of circulatory distress and oxygen deficiency of organs that includes ischaemia, organopathy, and a high risk of local and systemic infections.^7^–^9^ Many therapeutic efforts are known to reduce the intravascular protein deficiency. Treatment options are mainly based on simple volume substitution during the first 24 hours posttrauma according to the burned surface area and the patient's body weight.^10^ These fluids may be supplemented with electrolytes, proteins, plasma expander, fresh frozen plasma (FFP), or clotting factors, beginning 24 hours posttrauma.^11^,^12^ However, proteins are administered without exact knowledge of the quantitative or qualitative need.

Although severe infections are the main cause of mortality in patients with severe burn injuries, only few data are available on the presence of immunoglobulins (Igs) in human burn wounds.^13^–^15^

This study was designed to provide qualitative and quantitative data on the amount of protein loss in second-degree human burn wounds. In addition, we compared the effectiveness of two different protein solutions: FFP and Biseko^©^.

## MATERIAL AND METHODS

### Patients

Forty patients with a burnt TBSA of 20% to 60% (32.36 ± 18.19%), ages 38 to 63 years (48.53 ± 7.58 years), were included in the study. Exclusion criteria included inhalation trauma, severe systemic illness (renal insufficiency, hepatic cirrhosis child B and C, symptomatic heart insufficiency NYHA II, and malignant diseases), infectious diseases (human immunodeficiency virus infection, hepatitis B/C), and alcohol or drug abuse.

Fluid resuscitation was calculated using the Parkland formula (4-mL Ringer/kg body weight/% burnt TBSA) and administered by needs of the Baxter formula (50% of the calculated volume administered in the first 8 hours and 50% in the last 16 hours of the first 24 hours posttrauma). None of the patients received colloidal infusions in the first 24 hours posttrauma. No surgical intervention was performed and no catecholamines were given during the first 48 hours posttrauma. None of the test persons died during the course of the study. All burn wounds, except the wound chamber area, were treated with flammazine (silver sulphadiazine) wound dressing.

### Colloid resuscitation

Protein solutions were not administered to any patient within the first 24 hours posttrauma.

Twenty patients received FFP and 20 patients received the protein solution Biseko^©^ in a prospective randomized matter. Equal volumes were administered to the groups, starting 24 hours posttrauma.

Biseko^©^ is an isotonic human protein solution. It contains proteins of the human serum in physiological composition and active form. One-liter Biseko^©^ solution contains 50-g total protein (TP), including 31-g AL, 7.1-g IgG, 1.55-g IgA, and 0.48-g IgM. It contains stable and biologically intact Igs. Therefore, half-life, metabolism, and elimination match those of native serum. Further ingredients are sodium ions (3.56 g), potassium ions (0.16 g), calcium ions (0.08 g), magnesium ions (0.02 g), chloride ions (3.65 g), and water for injections.

The administered volumes were determined according to the Slater-formula (75 mL/kg/24 hours).^16^ Although the volume of protein substitution was calculated, the volume infused was titrated to maintain an adequate urine output (minimum 0.5–1 mL/kg/hour).

### Chamber design

**Figure 1:**
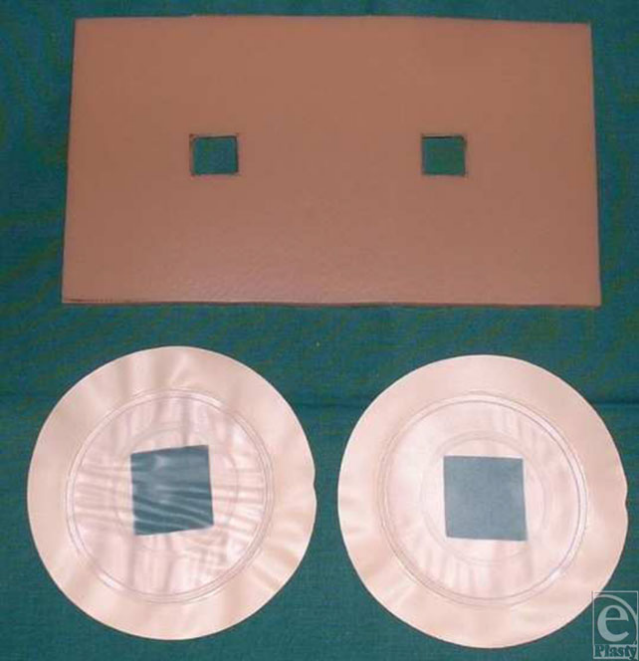
Silicon plate and vinyl chambers. The applied chamber system. Yellow coloring provides the protein content.

Two wound chambers (TMED, Inc, Columbia, Tenn) were placed on silicon plates (14×9 cm) with two 2.25 cm^2^ openings (1.5×1.5 cm^2^) as previously described^17^ (Fig 1a). After cleansing and disinfection, silicon plates and chambers were fixed to the center of the burn wound using Enbucrilat (Histoacryl, Braun Medical AG, CH-6020 Emmenbrücke) and filled with 2.5 mL of 0.9% sodium chloride solution. A 14×9 cm^2^ second-degree burnt area on the anterolateral thigh was enclosed in the cutaneous vinyl chamber system. Each patient received two chambers on one silicon plate on the wounded skin and one chamber placed on unwounded skin as control. Chambers were placed 2 to 4 hours posttrauma. The accumulated wound fluid (WF) was harvested with a 10-mL syringe and a 20-gauge needle every 8 hours and replaced by 2.5 mL of saline for a total time period of 48 hours until eschar excision was performed. As in WF sampling, serum was gained from central venous catheters (10-mL Serum Monovette® BraunC AG, Frankfurt, Germany).

The study was approved by the ethical committee of the Ruhr-University (Bochum, Germany) (registration No. 1516), and written consent was given by each patient or a legal representative.

### Collection of WF and blood samples

Wound fluid was harvested in 8-hour intervals by puncture of the chambers' superstructure. The consistency of the WF varied from transparent liquid to fibrinoid viscous.

All samples were centrifuged at 2000×*g* for 9 minutes at 4°C to separate the cells from fluid. The supernatant was partitioned in aliquots, shock frozen in liquid nitrogen, and stored at ‐82°C. Samples were analyzed for TP content, AL, and the Igs A, E, G, and M. At various time points, blood samples were analyzed for the inflammatory parameters—c-reactive protein (CRP) and white blood cell—and the coagulation parameters—prothrombin time, partial thromboplastin time, antithrombin concentration, and, finally, the fibrinogen concentration.

### Analysis of proteins

Serum TP content was measured with the biuret method (Synchron LX-System, Beckmann Coulter, Inc, Krefeld, Germany). Serum AL was determined by the Bromcresolpurpur method (Synchron LX-System, Beckmann Coulter, Inc). IgG, M, and A were defined nephrelometric (Immage Immunchemisystem, Beckmann Coulter, Inc). IgE was measured with an immunoassay (Unicap, Pharmacia Diagnostics, Freiburg, Germany). Wound fluid TP content was determined with the pyragallol red method (CX-9, Beckmann Coulter, Inc). Wound fluid AL content was detected nephrelometric (Immage Immunchemisystem, Beckmann Coulter, Inc).

### Analysis of the biopsies

To verify the severity of the thermal injury and to validate the burn depth, biopsies were taken at the edge of the silicon chambers. Next to the area of chamber placement, a cutaneous biopsy of 1×1 cm^2^ was taken and analyzed. The biopsies were analyzed histologically by haematoxylin-eosin- or Hinshaw-Pearse-staining (Department of Pathology at the BG University Hospital Bergmannsheil Bochum) (data not shown).

### Calculation of TBSA and protein quantification in serum

Protein quantification in WF was performed by a division of measured concentrations with the appropriate chamber volume. Because of the standard of determining the patient's burnt skin as a percentage of TBSA, the TBSA of each patient was calculated using the DuBois formula, which is widely used for TBSA prediction (eg, in chemotherapy).^18^,^19^ The measured amount of protein per chamber was converted into the amount of protein per 10% burnt TBSA.

Plasma volume was calculated by means of the Retzlaff equation, with which plasma volume in relation to height, weight, and haematocrit of patients with normal and pathological haematocrit can be accurately determined.^20^

## RESULTS

High values of the measured protein concentrations were detected at all time points in the burn wounds. There was a marked decrease of all detected protein contents in the serum that was contrary to WF concentrations.

### Chamber volume

Each wound chamber contained 3.11 ± 1.13 mL of WF on average after an 8-hour interval posttrauma, varying from 2.5 mL to 5.8 mL.

### Total protein

Mean TP concentration in wound chambers was 0.93 ± 0.43 g/dL. According to the correlative chamber volume, each chamber contained 0.02 ± 0.008 g of protein. Analogous to a 10% burnt TBSA, this is equivalent to a protein loss of 16.6 ± 8.86 g in a collection period of 8 hours.

**Figure 2:**
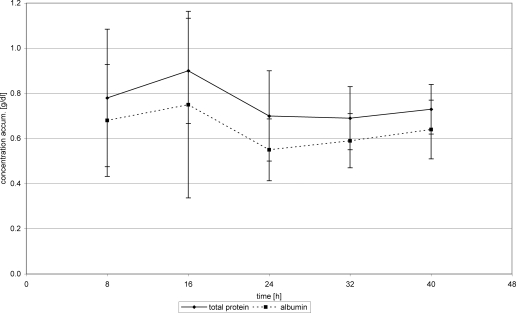
Total protein and albumin in wound fluid. Protein substitution started 24 hour posttrauma using fresh frozen plasma.

**Figure 3:**
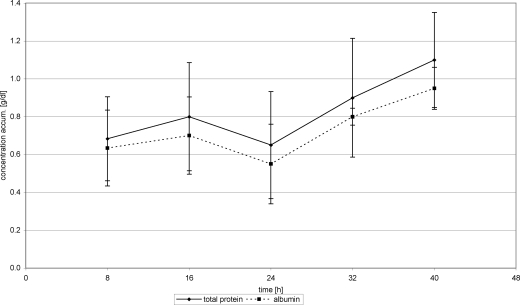
Total protein and albumin in wound fluid. Protein substitution started 24 hour posttrauma using Biseko.

With the introduction of FFP substitution 24 hours posttrauma, WF protein concentration nearly stagnated at about 0.7 g/dL (24 hours) (0.73 g/dL 40 hours) (Fig 2). Substitution of Biseko^©^ resulted in an increase of WF protein concentration from 0.65 g/dL after 24 h to 1.1 g/dL from after 40 hours posttrauma (Fig 3).

**Figure 4:**
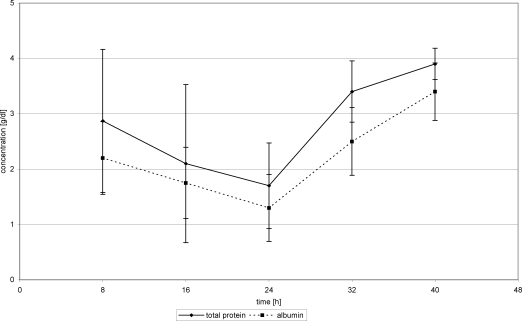
Total protein and albumin in serum. Protein substitution started 24 hour posttrauma using fresh frozen plasma.

**Figure 5:**
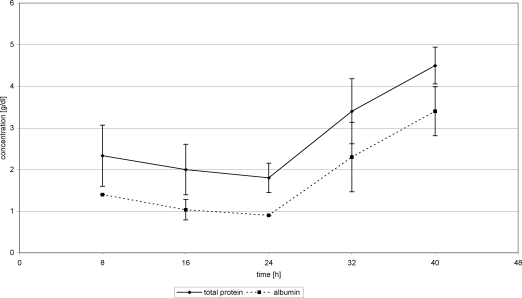
Total protein and albumin in serum. Protein substitution started 24 hour posttrauma using Biseko.

Total protein concentrations in serum in the first 24 hours posttrauma decreased in all patients. After beginning the protein administration, TP concentrations began to rise. On day 2 posttrauma, serum protein concentrations increased from 1.7 g/dL (24 hours) to 3.9 g/dL (40 hours) (Fig 4). Substitution of Biseko^©^ resulted in an even stronger rise of serum protein concentration from 1.8 g (24 hours) to 4.5 g (40 hours) (Fig 5).

## Albumin

Overall mean AL concentration in WF was 0.62 ± 0.2 g/dL in the first 24 hours, which is equivalent to 0.019 ± 0.007 g per chamber, and 12.39 ± 5.87g on a 10% burnt TBSA. This corresponds to an AL loss of 38.44 g in 10% burnt TBSA in 24 hours posttrauma. The changes of AL values in WF were comparable with those of TP in WF (Figs 2 and 3).

FFP substitution resulted in an AL concentration increase in WF from 0.55 g/dL (24 hours) to 0.64 g/dL (40 hours), whereas substitution of Biseko^©^ resulted in a stronger increase of the AL concentration from 0.55 g/dL (24 hours) to 0.95 g/dL (40 hours) (Figs 2 and 3).

During FFP substitution, AL concentration in serum increased from 1.3 g/dL (24 hours) to 3.4 g/dL (40 hours) (Fig 4). In the Biseko^©^ group, the starting values were lower those in the FFP group, 0.9 g/dL (24 hours), but after the addition of Biseko^©^ the AL concentration rose to similar values 3.4 g/dL (40 hours) compared with that in the FFP group (Fig 5).

## Immunoglobulins

The highest concentrations of Igs in WF and serum were found for IgG (WF: 157.09 ± 88.58 mg/dL; serum: 396.62 ± 216.54 mg/dL), whereas the lowest concentrations were found for IgA (WF: 57.7 ± 12 mg/dL). Correlated to 10% burnt TBSA, there was a 0.96-g mean loss of IgG within 8 hours posttrauma (16.5 g in 24 hours).

**Figure 6:**
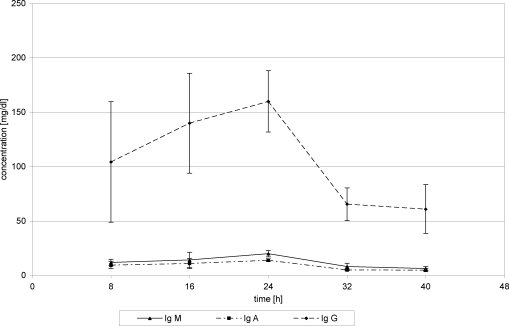
Immunoglobulins in wound fluid. Protein substitution started 24 hour posttrauma using fresh frozen plasma.

**Figure 7:**
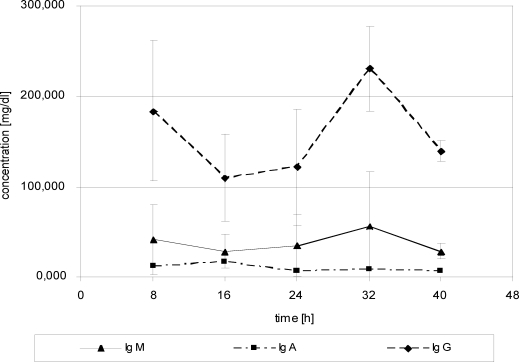
Immunoglobulins in wound fluid. Protein substitution started 24 hour posttrauma using Biseko.

Immunoglobulins in serum decreased until 24 hours postadmission and then stagnated with FFP substitution, except IgG, which revealed continuously decreasing values until the end of the measurements after 48 hours (Fig 6). In WF, IgG initially increased, while IgA and IgM revealed constant or decreasing values (Fig 7). After 24 hours, all Igs showed continuously decreasing values during FFP substitution (Fig 7).

**Figure 8:**
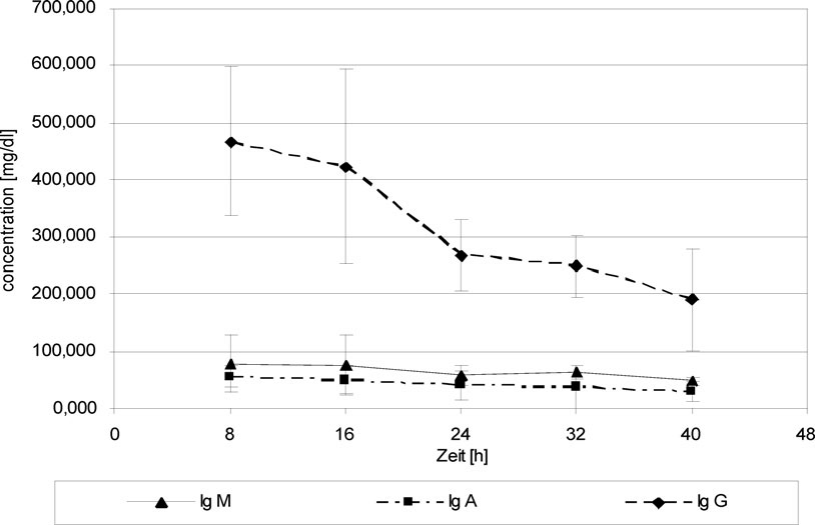
Immunoglobulins in serum. Protein substitution started 24 hour posttrauma using fresh frozen plasma.

**Figure 9:**
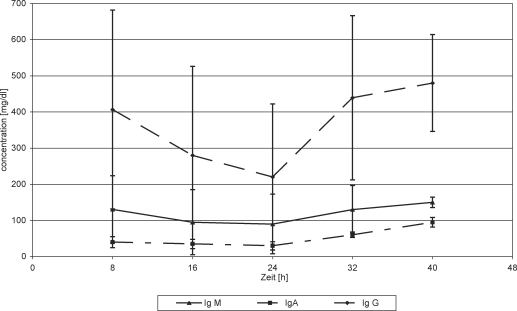
Immunoglobulins in serum. Protein substitution started 24 hour posttrauma using Biseko.

After Biseko^©^ substitution, IgG, IgM, and IgA showed increasing values in serum, whereas IgG revealed the highest rise (Fig 8). Wound fluid Igs values, especially IgG, increased after Biseko^©^ substitution (Fig 9).

## Coagulation parameters

**Figure 10:**
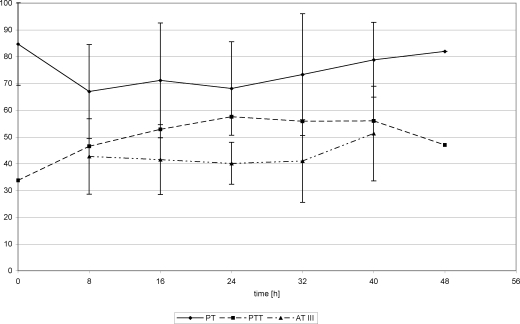
Coagulation parameters in serum. Protein substitution started 24 hour posttrauma using fresh frozen plasma.

**Figure 11:**
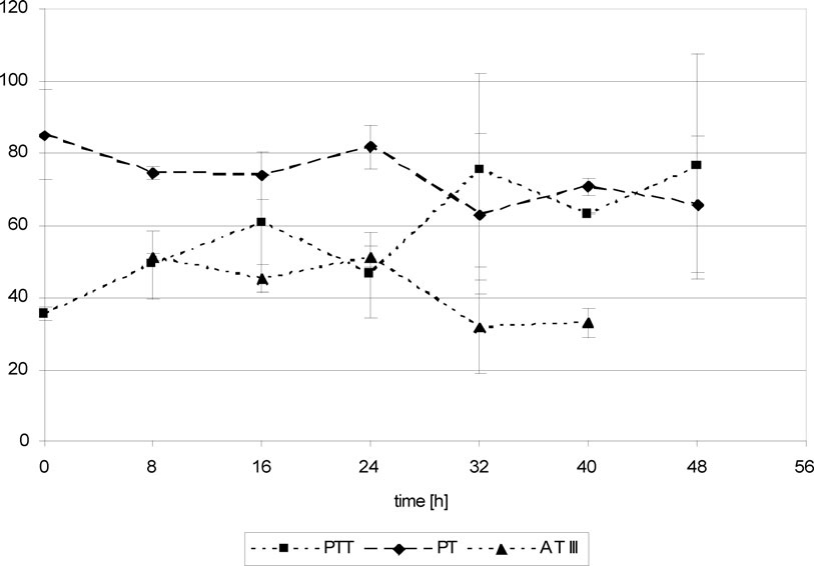
Coagulation parameters in serum. Protein substitution started 24 hour posttrauma using Biseko.

**Figure 12:**
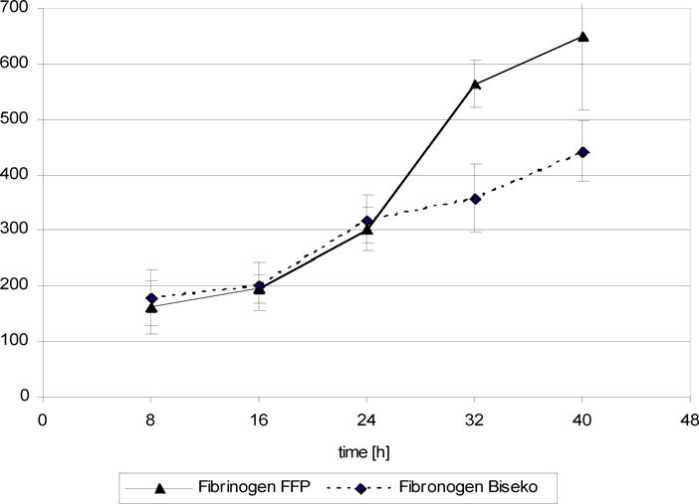
Fibrinogen in serum. Protein substitution started 24 hour posttrauma using fresh frozen plasma or Biseko.

Twenty-four hours posttrauma, fibrinogen concentration showed increasing values in both groups, whereas the remaining parameters revealed inconsistent progression (Figs 10–12). After FFP substitution, PT and PTT values remained with insignificant changes while AT III and fibrinogen values slightly increased (Figs 10 and 12). After Biseko^©^ substitution at 24 hours, fibrinogen concentration and PTT had increased significantly (Figs 11 and 12).

## Inflammation parameters

**Figure 13:**
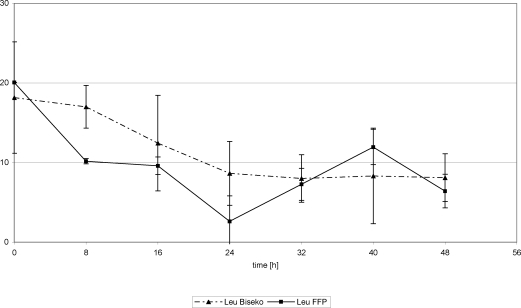
C-reactive protein and leucocytes in serum. Protein substitution started 24 hour posttrauma using fresh frozen plasma.

**Figure 14:**
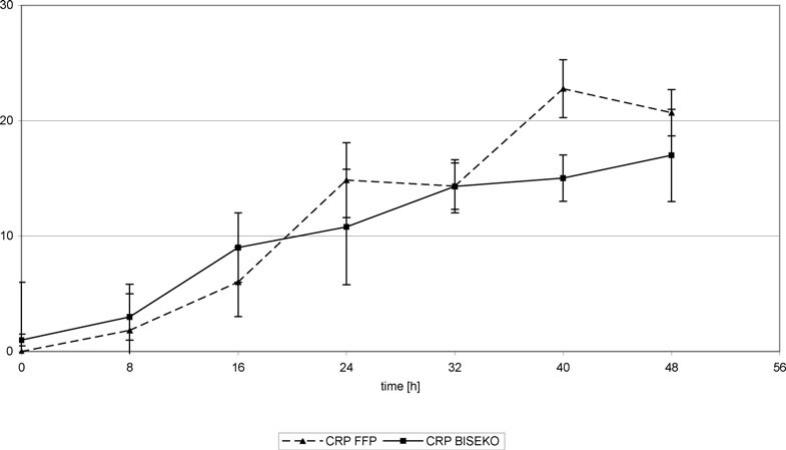
C-reactive protein and leucocytes in serum. Protein substitution started 24 hour posttrauma using Biseko.

Serum CRP levels continuously increased and showed similar values in both groups up to 32 hours posttrauma (Ø 8.5 mg/dL). While CRP peak-concentrations in the FFP group was found 40 hours posttrauma (23 mg/dL), in the Biseko^©^ group, peak value was found 48 hours posttrauma (17 mg/dL) (Figs 13 and 14).

White blood cells showed similar values. Beginning from an initial peak in both groups (Ø 19/nL), there was a decrease detectable up to 24 hours posttrauma in both the FFP and Biseko^©^ groups. From that time point, a plateau was seen in the Biseko^©^ group (Ø 7.5/nL up to 48 hours posttrauma) and a significant increase in the FFP group (Ø 13/nL at 48 hours posttrauma) (Figs 13 and 14).

## Biopsies

All biopsies taken confirmed deep partial burnt skin (data not shown). No proteins could be detected over unwounded skin.

## DISCUSSION

In patients suffering from burn wounds with more than 15% TBSA, disorders in several systems (eg, immune, vascular, and electrolyte system) result in a severe and unique derangement called burn shock.^11^ The pathophysiology of the burn shock manifests with the full spectrum of the complexity of the inflammatory response.^17^,^21^,^22^

Initially, burn shock is hypovolemic in nature and is characterized by hemodynamic changes similar to those that occur after hemorrhage, including decreased plasma volume, cardiac output, urine output, and an increased systemic vascular resistance, resulting in reduced peripheral blood flow.^23^ The loss of intravascular proteins results from CL syndrome and is induced by an endothelial dysregulation, including increasing endothelial permeability, hypermetabolism, protein catabolism, disseminated intravascular coagulation, and the direct destruction of local proteins in the region of heat impact.^1^,^24^

Capillary leakage represents the end of a cascade that is initiated and influenced not only by different mediators like histamine, prostaglandins, thromboxane, kinins, serotonin, catecholamines, and oxygen radicals but also by direct mechanical stress resulting in increasing intercellular clefts caused by in- and extracellular swelling.^4^ It takes place in the region of heat impact as well as in other body regions.^24^ In heat-damaged body parts, several different factors are responsible for the dispersion of circulating volume with resulting edema, such as local destruction of endothelial continuity, local denaturation and loosening of interstitial proteins, local inflammatory reaction, and CL.^1^,^25^–^27^ In body parts not damaged by heat, the oncotic pressure is most likely the main factor in the ensuing volume shift to interstitial tissue; therefore, intravascular plasma proteins are extremely important because they can decrease the extravasation of the circulating fluids.

### Time of colloid resuscitation

Consequently, protein replacement was an important component of early formulas for burn management.^28^ Considerable confusion exists concerning the role of proteins in a resuscitation formula and is still a matter of discussion. There are mainly three schools of thought:

1. Protein solutions should not be given within the first 24 hours: they are not effective in resuscitation because of shifting to the interstitium and they promote accumulation of lung water when edema fluid is being absorbed from the burn wound.^12^,^29^

2.Proteins should be given from the beginning of resuscitation, along with crystalloid, to counter the protein deficiency and its disadvantages.^30^

3. Protein should be given after 8–12 hours postburn; resuscitation should be strictly crystalloid in the first 8–12 hours because of the massive extravasation of fluids during this period.^7^

This study demonstrates an average wound protein loss of 16.6 ± 8.86 g in a collection period of 8 hours in a 10% burnt TBSA. Total protein values measured in wound chambers were consistently high until the end of measurements. These findings confirm results of a previous study in which a patient's TP amount in serum is lost to a wound surface of 20% burnt TBSA in 24 hours.^31^ Substitution of FFP and Biseko^©^ beginning 24 hours posttrauma rises the intravascular protein and AL concentration and consecutively increases intravascular oncotic pressure. Furthermore, TP and AL concentration also increases in WF and reveals a persistent leakage of proteins for a time period of more than 24 hours posttrauma. The data suggest that the CL persists significantly longer than the estimated time of 24 hours posttrauma; however, the protein substitution results in increasing serum values of TP and AL in both the FFP and Biseko^©^ groups and, furthermore, increasing values of Igs in the Biseko^©^ group. The resulting increased intravascular oncotic pressure and the immune status might be beneficial for the patient. We therefore recommend an early substitution beginning after 24 hours posttrauma although the CL persists.

### Type of resuscitations solutions

The choice of the type of protein solution in burn injuries can also be confusing. After 24 hours posttrauma, several add-ups, for example, plasma expander and colloidal or AL solutions, are administered.^32^,^33^ Several different strategies for plasma volume expansions are used: for example, FFP, AL, starches, gelatins, dextrans, and different human protein compilations. At present, in most burn centers, physicians use 5%–20% AL in isotonic crystalloid solution or FFP to increase the intravasal oncotic pressure, whereas gelatins are less common and dextrans are only rarely used.^34^ Standard protein supplements result in increased concentrations of AL and, consequently, TP. Besides the singular colloidal supplements, protein compilations are used to elevate not only the AL and TP concentrations but also the Igs concentrations.

There are a few drawbacks that have to be accepted in using FFP. Beside its limited availability, the cold chain may not be interrupted. FFP has a limited durability (2 years including 4–6 months of quarantine). Before using, FFP must be thawed. A blood group–adapted infusion and documentation, as well as a bedside test, are essential. Furthermore, as a product of human blood, it has a marked risk of viral and bacterial infections although several technologies for pathogen reduction of plasma reduce the risk of bacteria and virus transmission.^35^,^36^ However, FFP's major advantage is still the content of haemostatic factors.

Biseko^©^ is a compilation of human AL and the Igs A, G, and M. It has a durability of 3 years and there is no need for a blood group–adapted infusion. Even though Biseko^©^ is a human product and there is consequently a risk of viral and bacterial infections, presently, no viral or bacterial infections after Biseko^©^ infusions have been documented.

This study reveals a considerable beneficial progression of serum-TP and AL concentrations in the Biseko^©^ group. Furthermore, we found an effective increase of the substituted Igs in the Biseko^©^ group.

Although Biseko^©^ does not contain coagulation parameters, there was a positive effect on blood coagulation in this group as well. A possible reason might be associated with the more physiological level of TP and AL. In consideration of the known multiple functions of AL, including its transportation functionality, the impact of high AL concentrations seems understandable.^37^

The registered systemic inflammatory parameters WBC and CRP showed lower values in the Biseko^©^ group. It shows varying immunological responses in the compared groups. The proteins administered do not interfere with the humoral-induced systemic inflammatory response syndrome or CL; nevertheless, there seems to be an effect of the proteins that leads to differing immunologic response.

In summary, commercial protein solutions like Biseko^©^ are valuable alternatives to pure AL or FFP substitution. The application might result in better conditions for the patient in view of starting with surgical interventions on day 3 posttrauma. Further trials with bigger patient groups are necessary to validate the beneficial aspects of different protein solutions.

## Acknowledgments

We thank Biotest-Pharma, Dreieich, Germany, for financial support and Amanda Daigeler for her formal English revision of the manuscript.
